# Medullary sponge kidney presenting in a neonate with distal renal tubular acidosis and failure to thrive: a case report

**DOI:** 10.1186/1752-1947-3-6656

**Published:** 2009-04-29

**Authors:** Mohamed El-Sawi, Abdul-Rahman Shahein

**Affiliations:** 1 Department of Medical Genetics, Ain Shams Medical School, 38 Abbassia, Cairo, Egypt; 2 Department of Pediatrics, University of Toronto, 555 University Ave, Ontario, Canada M5G 1X8

## Abstract

**Introduction:**

Medullary sponge kidney is a congenital anomaly characterized by diffuse ectasy of the collecting tubules of one or both kidneys. It is usually diagnosed in the second or third decade of life.

**Case presentation:**

Distal renal tubular acidosis is commonly observed in patients with medullary sponge kidney. We describe here a 50-day-old Egyptian Caucasian girl with medullary sponge kidney who had features of distal renal tubular acidosis, (persistent alkaline urine, hypercalciuria, hypocitraturia) and failure to thrive. Renal ultrasound revealed left renal increased medullary echogenicity and bilateral nephrocalcinosis.

**Conclusion:**

Early gene(s) expression of medullary sponge kidney disease might be responsible for persistent metabolic acidosis during the neonatal period.

## Introduction

Medullary sponge kidney (MSK) is a rare developmental abnormality characterized by cystic dilatation of the collecting tubules in one or more renal pyramids in one or both kidneys. Its precise prevalence is not known. Moreover, its pathogenesis is unknown but most authors agree that it is a congenital anomaly with delayed expression [1]. On the other hand, familial forms have also been described and the dominant mode of transmission has been proposed. Medullary sponge kidney is usually diagnosed in people aged 10 to 30 years on the basis of laboratory and radiological findings [2]. Discovery of a responsible gene(s) would be a great step forward in understanding the disease.

## Case presentation

A 50-day-old Egyptian Caucasian girl was referred to our department for persistent vomiting, dehydration and disturbed conscious level. The patient was born full term, 3500 kg (+1 SD) by normal vaginal delivery, with a cephalic presentation. Her mother is 26 years old, G3P3 with no family history of renal diseases and her husband is a first cousin. The mother reported the death of her second offspring, a girl, at the age of 3 months due to fever, vomiting and diarrhea. The condition appeared to be gastroenteritis but no medical or laboratory records are available. The first offspring of the parents is a 4-year-old healthy living girl.

Our patient's complaint started with persistent vomiting and dehydration when she was 15 days old. She was admitted to the neonatal intensive care unit (NICU) for 4 days with no significant improvement. Soon after discharge, her mother noticed an increase in her child's rate of breathing and presented to the medical facility again. Only serum ammonia was available during this period: 109 μmol/L (N: < 81 μmol/L).

At the age of 45 days, the patient's conscious level deteriorated, suckling decreased and she was admitted to the pediatric intensive care unit (PICU). On admission, her modified Glasgow Coma Scale (GCS) was 12, she had severe dehydration with dry mucous membranes, poor perfusion (capillary refill: 5 s), a temperature of 38.2°;C, weight 3300 kg (-2.8 SD) and bilateral diffuse sonorous rhonchi over her chest. Her complete blood count showed mild absolute neutrophilia with neutrophils (NE) 63%, C-reactive protein (CRP) 24 mg/dL (N < 6), chest anteroposterior (AP)/lateral X-ray images were free from pneumonic patches, serum creatinine 1.1 mg/dL (N: 0.3 to 1.0), and blood urea nitrogen (BUN) 31 mg/dL (N: 7 to 22). Her chest infection and dehydration were successfully treated with third generation cephalosporins and intravenous fluids.

Our patient was then strongly considered to have distal renal tubular acidosis (dRTA) based on the presence of the following: failure to thrive, growth retardation, hyperchloremic metabolic acidosis with respiratory alkalosis and persistent alkaline urine (pH > 7). Urine and blood tests were performed to confirm the diagnosis and distinguish between proximal and distal renal tubular acidosis (Table 1).

**Table 1:** Laboratory evaluation of the patient on admission

Tests	Results
**Urine**	
Specific gravity	1008
pH	8
Glucose	Normal
Proteins	Normal
Aminogram	Normal
Citrate	1.2 mg/kg/day (N > 2.0)
Calcium	12.3 mg/kg/day (N < 5.0)
**Blood**	
pH (Venous)	7.28 (N: 7.35-7.45)
PCO_2_ (Venous)	25.8 mmHg (N: 37-47)
HCO_3_^-^ (Venous)	11.9 mmol/L (N: 21-28)
Na	132 meq/L (N: 138-145)
K	1.4 meq/L (N: 3.5-5.0)
Cl	113 meq/ (N: 96-106)
Uric acid	2.9 mg/dL (N: 2.0-5.5)
Ca	9.7 mg/dL dL (N: 8.8-10.8)
P	3.1 mg/dL (N:4.5-5.5)
Serum anion gap	8
Serum NH_3_	58.19 μmol/L (N: < 81 μmol/L)
	

**Figure 1:**
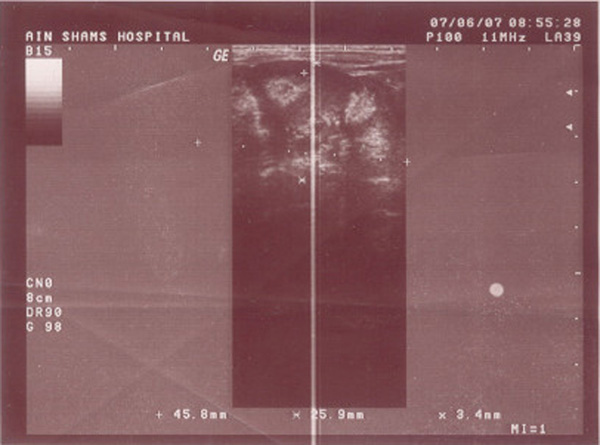
**Left kidney ultrasound showing nephrocalcinosis and hyperechoic renal medulla; spongy appearance**.

**Figure 2:**
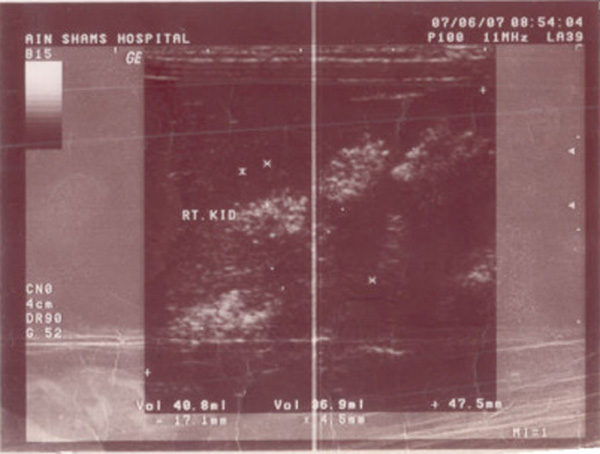
**Right kidney ultrasound showing diffuse nephrocalcinosis**.

**Figure 3:**
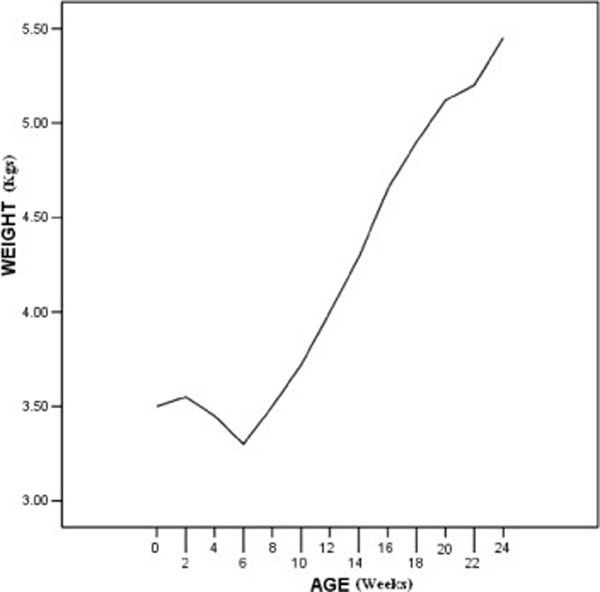
**Growth curve; patient growth dramatically improved after correction of electrolyte deficiency**.

She started to receive intravenous bicarbonate infusion and potassium supplementation with partial response. Pelvi-abdominal ultrasound (U/S) revealed bilateral nephrocalcinosis and left renal increased medullary echogenicity with a picture suggestive of medullary sponge kidney (Figures 1 and 2). She had no abnormality in auditory brainstem response and in a skeletal survey. After 1 month of treatment with oral bicarbonate 3 meq/kg/day, KCl 2 meq/kg/day, patient vomiting and weight improved. The last laboratory tests were as follows: pH 7.435 (N: 7.35 to 7.45), HCO_3_^-^ 20.9 mmol/L (N: 21 to 28), PvCO_2_ 29.6 mmHg (N: 37 to 47), Cl^-^ 110 meq/L (N: 96 to 106), Na^+^ 134 meq/L (N: 138 to 145), K^+^ 3.9 meq/L (N: 3.5 to 5.0), serum creatinine 0.3 mg/dL (N: 0.3 to 1.0). Her growth over 6 months of follow-up after treatment showed a marked improvement (Figure 3).

## Discussion

Our patient clearly developed a significant hyperchloremic metabolic acidosis with respiratory alkalosis and normal anion gap during her neonatal period associated with recurrent infection, failure to thrive and renal nephrocalcinosis. The presence of hyperechoic medulla in her left kidney raised the diagnosis of medullary sponge kidney as an underlying cause (Figure 1).

Although the above sonographic appearance of MSK is non-specific, the degree of confidence of ultrasound in diagnosing MSK is still superior to other radiological techniques [3,4]. Hyperechoic medulla with or without shadowing has been documented in gout, Sjögren syndrome, systemic lupus erythematosus, hyperparathyroidism [5], glycogen storage diseases, Wilson disease, primary aldosteronism, and pseudo-Bartter syndrome [6]. Yet these etiologies were excluded in our patient both clinically and from the results of laboratory tests. Intravenous pyelography (IVP) is another radiological measure of high value in diagnosing MSK, but it was refused by her parents.

Patient growth curves were delayed and she suffered from failure to thrive (Figure 3). Moreover, after resuming proper feeding, her growth velocity remained below normal levels for the following 6 months. Sluysmans *et al.*[7] reported a 12-year-old girl with medullary sponge kidney and failure to thrive who responded on alkali therapy.

To our knowledge, there is no previously published work about MSK associated with dRTA as a cause of persistent metabolic acidosis during the neonatal period. Medullary sponge kidney is usually diagnosed in the second or third decade of life due to delayed expression of the gene(s) responsible for this anomaly, although Belde *et al.*[8] reported a 5-year-old girl with MSK and growth retardation. This may indicate the possibility of early gene(s) expression in MSK.

The expected renal outcome in MSK is excellent as long as urinary tract infections and nephrolithiasis can be prevented [9]. Although significant renal impairment is uncommon for this disorder, Pesce *et al.*[10] reported a child with bilateral medullary sponge kidney and chronic renal insufficiency.

Fewer than 5% of cases are familial and a clear genetic basis for medullary sponge kidney has not been established. The only genetic pattern observed in select pedigrees is an autosomal dominant type of transmission 2,[11,12].

## Conclusion

Medullary sponge kidney associated with dRTA should be considered in neonates and may indicate the possibility of very early expression of the genetic disease.

Simple oral alkali therapy is sufficient to treat some metabolic disorders associated with renal tubular dysfunction.

## Abbreviations

AP: anteroposterior; BUN: blood urea nitrogen; CRP: C-reactive protein; PICU: pediatric intensive care unit; NICU: neonatal intensive care unit; GCS: Glasgow Coma Scale; IVP: intravenous pyelography; N: normal; dRTA: distal renal tubular acidosis; U/S: ultrasound; meq: mille equivalent; PvCo_2_: partial pressure of venous carbon dioxide; MSK: medullary sponge kidney; NE: neutrophils.

## Consent

Written informed consent was obtained from the father of the patient for publication of this case report. A copy of the written consent is available for review by the Editor-in-Chief of this journal.

## Competing interests

The authors declare that they have no competing interests.

## Authors' contributions

ME interpreted the patient data regarding the genetic inheritance of medullary sponge kidney. AS analyzed and interpreted the patient's clinical presentation regarding renal disease associated with medullary sponge kidney. All authors read and approved the final manuscript.
